# A new mechanism for dendritic pattern formation in dense systems

**DOI:** 10.1038/srep28960

**Published:** 2016-06-29

**Authors:** Noriko Oikawa, Rei Kurita

**Affiliations:** 1 Department of Physics, Tokyo Metropolitan University, Tokyo 192-0397, Japan

## Abstract

Patterns are often formed when particles cluster: Since patterns reflect the connectivity of different types of material, the emergence of patterns affects the physical and chemical properties of systems and shares a close relationship to their macroscopic functions. A radial dendritic pattern (RDP) is observed in many systems such as snow crystals, polymer crystals and biological systems. Although most of these systems are considered as dense particle suspensions, the mechanism of RDP formation in dense particle systems is not yet understood. It should be noted that the diffusion limited aggregation model is not applicable to RDP formation in dense systems, but in dilute particle systems. Here, we propose a simple model that exhibits RDP formation in a dense particle system. The model potential for the inter-particle interaction is composed of two parts, a repulsive and an attractive force. The repulsive force is applied to all the particles all the time and the attractive force is exerted only among particles inside a circular domain, which expands at a certain speed as a wave front propagating from a preselected centre. It is found that an RDP is formed if the velocity of the wave front that triggers the attractive interaction is of the same order of magnitude as the time scale defined by the aggregation speed.

Patterns are ubiquitously observed in a whole myriad of systems e.g. biological systems, phase separation, gelatines, surfactants and block copolymers^1^,^2^,^3^,^4^. The patterns often vary the macroscopic properties of the system and give the systems functionality. For example, network patterns in solid-gas foams not only have the benefit of reducing weight, but impart shock resistance to the system; it is also known that the conductivity suddenly increases when a network of conducting particles percolates the system. Radial dendritic patterns (RDPs) are observed in particle systems such as colloidal dispersions and cell populations. We note that dendritic patterns have the advantage of allowing a large number of dispersed particles to cohere together. That is, even very distant particles can connect and the resulting cluster spreads over a wide area. When conducting particles form RDPs, the system becomes conductive over a wide range. The social amoebae *dictyostelium discoideum* also form an RDP when they aggregate^5^,^6^,^7^,^8^,^9^,^10^,^11^. Thus the RDP is clearly one of the most important patterns for system function, and understanding the mechanism of RDP formation is expected to lead to useful applications.

The diffusion limited aggregation (DLA) model is known as a mechanism for RDP formation in dilute particle systems. In the DLA model, diffusing particles randomly collide and stick to each other, eventually forming a radially expanded dendritic structure^12^,^13^. However, the DLA model is not applicable to RDP formation in dense systems. When an RDP forms in dense systems, it is sometimes observed that many small clusters are generated first and the dendritic pattern grows successively via aggregation of the clusters. Thus, the formation process of RDPs in dense particle systems should be distinguished from that of dilute systems: the mechanism for RDP formation in dense systems has not yet been clarified.

In this Letter, we introduce a simple model that creates an RDP in a dense particle system. The model is based on a potential that combines an attractive and a repulsive force. The spatial extent of the region in which the attractive interaction is applied between particles is limited to a circular domain which expands at a certain speed as a wave front propagating from a preselected central point. The speed of the expansion of the domain edge, hereon referred to as the triggering wave front, is an important parameter for RDP formation. It is demonstrated that the model produces RDPs in dense particle systems when the velocity of the triggering wave front is of the same order of magnitude as the aggregation time scale. Due to the simplicity of the proposed model, the model is expected to provide a general mechanism for the formation of radial dendritic patterns observed in many systems.

## Model

The particles move as a result of attractive and repulsive interactions with other particles. The repulsive force is applied to all the particles all the time. The attractive force is exerted only among particles inside a circular domain, which expands at a certain speed as a wave front propagating from a preselected point. We refer to the circular boundary of this domain as the triggering wave front. The origin of the triggering wave source, from which the triggering wave front propagates, is preselected, and the core of the RDP that eventually forms is located at the same point. The attractive interaction occurs if *r*_*i*_ ≤ *r*_*tri*_(*t*), where *r*_*i*_ is the distance from the source to a particle *i* and *r*_*tri*_(*t*) is the distance from the source to the trigger wave front. The total potential energy of the particles is defined as 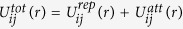, if particle *i* and particle *j* are located inside the triggering wave front, and 
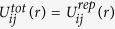
 otherwise, where 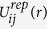 and 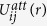 are the potential energies for the repulsive force and the attractive force between the particle *i* and the particle *j*, respectively. For our numerical simulations, we used the Weeks-Chandler-Andersen potential for the repulsive interaction and a Van-der-Waals potential for the attractive interaction (see Methods). Unlike the DLA model, thermal noise is neglected, that is, the particles do not diffuse with time. We also exclude hydrodynamic interactions for simplicity. The key feature of the present model is the existence of a domain region inside which the attractive interaction is effective, rather than the type of the potential.

## Numerical Simulations

**Figure 1:**
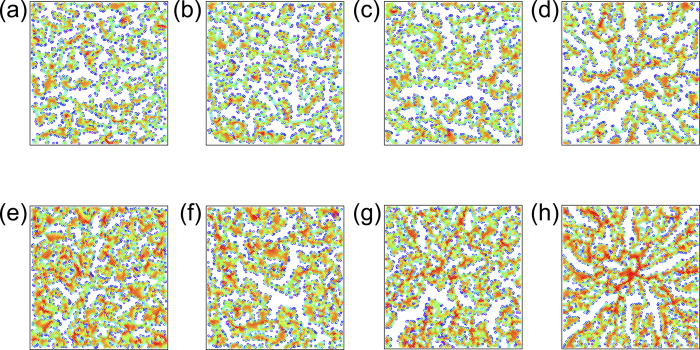
Asymptotic states of the aggregation pattern for *v*_*tri*_ = ∞ (**a**), 10^−1^ (**b**), 10^−2^ (**c**) and 10^−3^ (**d**) in *ϕ* = 0.60, and for *v*_*tri*_ = ∞ (**e**), 10^−1^ (**f**), 10^−2^ (**g**) and 10^−3^ (**h**) in *ϕ* = 0.78. Colours assigned to particles represent the value of the potential energy. Red and blue correspond to lower and higher energy, respectively.

We perform numerical simulations for the model while changing the area fraction *ϕ* occupied by the particles and the propagation speed *v*_*tri*_ of the triggering wave front. The calculation has been performed for two particle numbers, *N* = 7000 and 9000, which correspond to *ϕ* = 0.60 and 0.78, respectively. The origin of the triggering wave source is set to be the centre of the simulation box. Figure 1 shows the asymptotic states of the aggregation patterns. Since there are no thermal fluctuations, the patterns are stable once they create a domain structure. The images (a) to (d) and (e) to (h) represent the results for *ϕ* = 0.60 and *ϕ* = 0.78, respectively. The values of *v*_*tri*_ used for the each image are (a) (e): ∞, (b) (f): 10^−1^, (c) (g): 10^−2^ and (d) (h): 10^−3^.

When *v*_*tri*_ is infinitely fast (*v*_*tri*_ = ∞), small clusters of particles are created (Fig. 1(a,e)). The aggregation speed of the particles is given by the distance the particle moves in one time step of the calculation divided by the time. The average value of the aggregation speed of the particles over the particles in a circular shell at the propagation front is defined as *v*_*agg*_. When *ϕ* = 0.78 (Fig. 1(e)), *v*_*agg*_ ∼ 2.05 × 10^−2^, and *v*_*agg*_ ≪ *v*_*tri*_. In this case, the attractive interaction is effective for all the particles from the beginning, regardless of the position of the particles. Therefore, the particles are locally gathered in the proximity of their initial positions, and small clusters are created. The situation is similar to that observed in the aggregation of colloidal particles in the absence of flow^14^. In Fig. 1(b,f), *v*_*tri*_ (=10^−1^) is still fast compared to the value of *v*_*agg*_ ∼ 1.61 × 10^−2^ and the clusters remain local and disordered. When *v*_*tri*_ is decreased to 10^−2^ (Fig. 1(c,g)), *v*_*tri*_ becomes the same order of magnitude as *v*_*agg*_ (∼6.64 × 10^−3^ for *ϕ* = 0.78). Under this condition, the clusters start to connect with each other and the size of the clusters becomes larger. With a further decrease of *v*_*tri*_ to 10^−3^ (Fig. 1(d,h)), the clusters form RDPs in which elongated clusters are spread from the centre of the triggering wave source. Under this condition for *ϕ* = 0.78, the value of *v*_*agg*_ is 1.75 × 10^−3^ at the propagation front and is comparable to the value of *v*_*tri*_.

**Figure 2:**
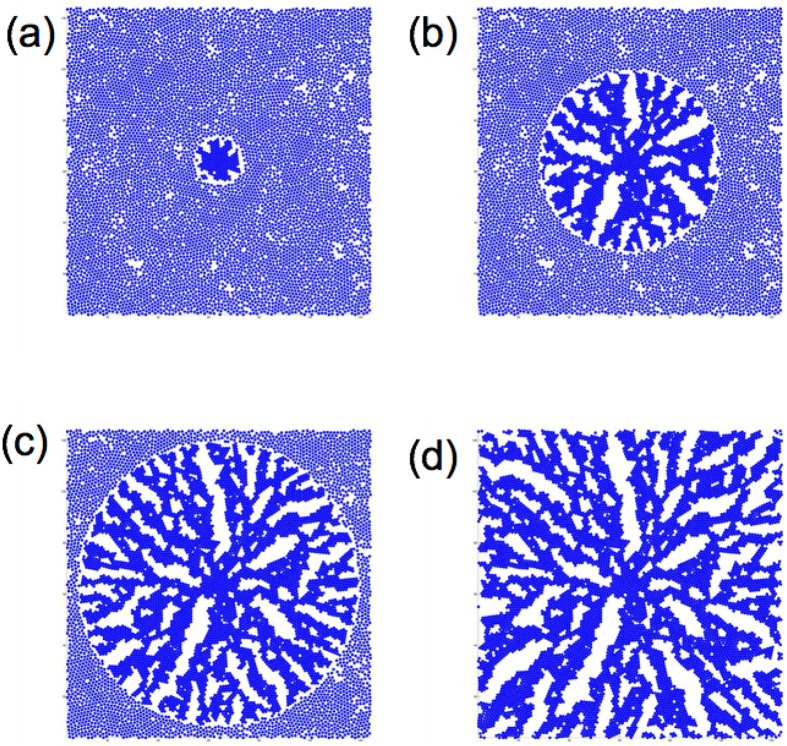
Time evolution of the aggregation patterns for *ϕ* = 0.78 and *v*_*tri*_ = 10^−3^ at (**a**) *t* = 1.0 × 10^4^, (**b**) 3.6 × 10^4^, (**c**) 5.6 × 10^4^ and (**d**) 8.3 × 10^4^.

In order to investigate RDP creation when *v*_*agg*_ ∼ *v*_*tri*_, the time evolution of the aggregation pattern is obtained. Fig. 2(a∼d) show snapshots of the pattern formation for *ϕ *= 0.78 and *v*_*tri*_ = 10^−3^ at *t* = 1.0 × 10^4^, 3.6 × 10^4^, 5.6 × 10^4^ and 8.3 × 10^4^. Since the triggering wave front propagates from the centre of the system, the particles start to aggregate in the central region, as seen in Fig. 2(a). The aggregate at the centre becomes the core of the RDP in the later stage, and it is noticed that the RDP grows from the core in the present system. The shape of the cluster becomes rough as the cluster grows due to spatial inhomogeneity in the initial arrangement of the particles, and branches are formed. While the particles tend to move towards the centre of the cluster since the density of the particle is higher at the centre, the attractive interaction acts more strongly between the nearest neighbour particles. Therefore, the particles in the region away from the centre attach to the tips of the branches of the cluster. As a result, the branches grow outwards, as shown in Fig. 2(b). In the final stage of the clustering process, an extended dendritic pattern appears as shown in Fig. 2(c). The dendritic pattern becomes slightly thicker with time and the aggregate cluster at the centre becomes larger, as shown in Fig. 2(d). The particles are eventually trapped at a local minimum of the potential energy and the growth of the pattern almost stops, since no thermal fluctuations are included in the present system. Since the RDP is formed via the process given above, the necessity of the condition *v*_*tri*_ ∼ *v*_*agg*_ seems to be reasonable for the formation of RDPs. (Please see Supplementary Information)

**Figure 3:**
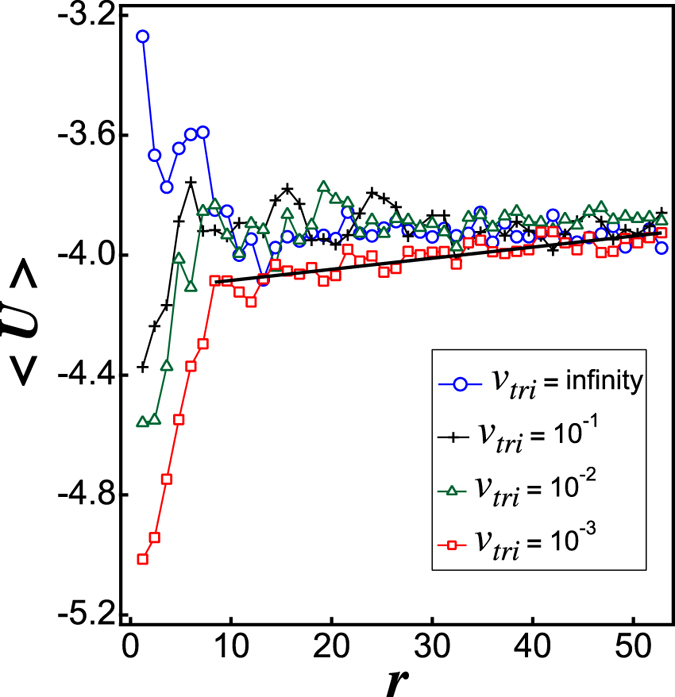
Averaged potential value 〈*U*〉 as a function of distance *r* from the centre for *v*_*tri*_ = ∞, *v*_*tri*_ = 10^−1^, *v*_*tri*_ = 10^−2^ and *v*_*tri*_ = 10^−3^. The solid line represents a linear fit to *v*_*tri*_ = 10^−3^.

To quantitatively analyse the asymptotic patterns, the potential energy is calculated for each particle and indicated by their colour in Fig. 1. Potential energy *U*_*i*_ of particle *i* is defined as 
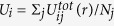
 for *r*_*ij*_ < 4*σ*, where *r*_*ij*_ and *σ* are the distance between particles *i* and *j* and their diameter, respectively. The colour assigned to each particle in Fig. 1 indicates the value of *U*_*i*_. Red and blue represent lower and higher potential energies, respectively. In (a) to (c) and (e) to (g) (*v*_*tri*_ = ∞ to 10^−2^), the potential minimums are distributed throughout the system. On the other hand, in (d) and (h) (*v*_*tri*_ = 10^−3^), it is clearly recognised that the potential minimum exists at the centre. The potential energy 〈*U*〉 averaged over the particles in a circular shell of the azimuthal direction is plotted as a function of the distance *r* from the centre in Fig. 3. The results are obtained by averaging the data for six different numerical simulations. In the lower *r* domain, it is recognised that 〈*U*〉 takes a small value except for in the *v*_*tri*_ = ∞ case. This indicates that a cluster is created in the centre where the triggering wave source is located, for *v*_*tri*_ = 10^−1^, 10^−2^ and 10^−3^. Since the triggering wave front propagates from the centre, the particles in the centre are able to generate an aggregate as long as *v*_*tri*_ has a finite value. The difference between RDPs and disordered clusters emerges in higher *r* regions, reflecting the nature of the latter stages of the growth process. In the higher *r* region, there is no dependence of 〈*U*〉 on *r* when *v*_*tri*_ = ∞, 10^−1^ and 10^−2^. For *v*_*tri*_ = 10^−3^, on the other hand, 〈*U*〉 gradually decreases towards *r* = 0, the centre of the RDP as shown by the fitting line plotted on 〈*U*〉 when *v*_*tri*_ = 10^−3^. Therefore, it is expected that, if the calculation is performed for a long time with thermal noise, the particles will move towards the centre for the *v*_*tri*_ ≤ 10^−3^ case, whereas when *v*_*tri*_ ≥ 10^−2^, the particles are trapped at the minimum energy points distributed throughout the system.

Here, we discuss the features of the present model in comparison with the DLA model. Firstly, in the DLA model, an RDP is only created in a situation where the number density of particles is low, while the present model shows RDP formation in quite dense systems. Secondly, the birth process of the core is different. The core of the RDP is created stochastically in the DLA model in a similar manner to the nucleation process for crystals in a supercooled liquid, whereas the core of the RDP is preselected; in other words, the position of the core can be controlled in the present model. Finally, diffusive dynamics is a dominant factor in the DLA model while RDPs can be observed without diffusion in the present model.

To summarise the paper, we proposed a simple model that produces radial dendritic patterns in dense particle systems. The inter-particle interaction energy for the particles is composed of a repulsive force which is applied to all the particles in the system and an attractive force which is only exerted among particles inside a domain region. The boundary of the domain expands at a certain speed from a preselected point. A radial dendritic pattern is formed when the speed of the domain boundary that triggers the attractive force is comparable to the intrinsic aggregation speed. Unlike the DLA model, the present model does not include diffusion and hydrodynamic interactions. Since the present model takes into account only a minimal number of factors, the speed *v*_*tri*_ of the triggering wave front and the area fraction *ϕ* occupied by the particles, it is expected that the mechanism for RDP formation can be applied to other systems, including real systems. The present model may provide a general mechanism for the formation of radial dendritic patterns in dense particle systems.

## Methods

We apply a Weeks-Chandler-Andersen potential for the repulsive interaction. 
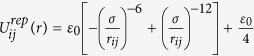
 if *r*_*ij*_ < 1.12 *σ*, otherwise 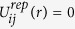, where *ε*_0_, *r*_*ij*_ and *σ* are the potential strength, the distance between particle *i* and particle *j* and the diameter of the particles, respectively. We also apply a Van-der-Waals potential for the attractive interaction with a cutoff length of 4 *σ*.  
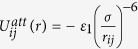
 if *r*_*ij*_ < 4 *σ* and both *r*_*i*_ and *r*_*j*_ < *r*_*tri*_(*t*), otherwise 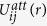 = 0. We set *ε*_0_ = 1 and *ε*_1_ = 2. We set *v*_*tri*_ to be constant for each numerical simulation. We normalise the length and the potential energy using *σ* and *ε*_0_. Time is scaled by 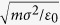.

## Additional Information

**How to cite this article**: Oikawa, N. and Kurita, R. A new mechanism for dendritic pattern formation in dense systems. *Sci. Rep.* **6**, 28960; doi: 10.1038/srep28960 (2016).

## Supplementary Material

Supplementary Movie 1

Supplementary Movie 2

Supplementary Information
