# Supplementary material for: A new mechanism for dendritic pattern formation in dense systems
Source: Sci Rep. 2016 Jun 29;6:28960. doi: 10.1038/srep28960 (PMC4926247; doi:10.1038/srep28960)
Supplement: Supplementary Information [file srep28960-s3.pdf]

Supplementary information

"A new mechanism for dendritic pattern formation in dense systems"

Noriko Oikawa and Rei Kurita

Department of Physics, Tokyo Metropolitan University, Tokyo 192-0397, Japan

Accompanying movies for Figure 1 (f) and (h) and Figure 2 showing temporal development of the aggregation patterns.

Supplementary video 1 (vtri10^-1): Time evolution of the aggregation pattern for $\phi = 0.78$ and $v_{tri} = 10^{-1}$ accompanying Figure 1 (f). The clusters formed are local and disordered.

Supplementary video 2 (vtri10^-3): Time evolution of the aggregation pattern for $\phi = 0.78$ and $v_{tri} = 10^{-3}$ accompanying Figure 1 (h) and Figure 2. The radial dendritic pattern is formed.
